# Supplementary material for: SARS-CoV-2 infection perturbs enhancer mediated transcriptional regulation of key pathways
Source: PLoS Comput Biol. 2023 Aug 10;19(8):e1011397. doi: 10.1371/journal.pcbi.1011397 (PMC10443870; doi:10.1371/journal.pcbi.1011397)
Supplement: Attachment — Submitted filename: rebuttal.v3.docx [file pcbi.1011397.s008.docx]

**RESPONSE TO REVIEWER COMMENTS**

We are very grateful for all the reviewers’ thoughtful comments and feedback. The reviewers made several suggestions, which we have now addressed in the revised manuscript with new analyses in most cases. Incorporating these suggestions has significantly improved the manuscript, and we look forward to sharing these findings more broadly with the scientific community.

Reviewer #1: 1. Title- It should be simple and clear. Consider modifying it.
2. Short Title- The present short title does not seem to convey the message of this study.

Thank you for pointing this out, we have simplified the title to “SARS-CoV-2 infection perturbs enhancer mediated transcriptional regulation of key pathways” and revised the short title to “SARS-CoV-2 infection perturbs regulation of key pathways”.

3. Keywords- Keep them specific. Avoid general words like ‘epigenetics’. Words from present title can be projected as keywords provided the title is considered to be modified. It would help to improve the citations of this study.

Thank you for the suggestion, we have revised the keywords to: COVID-19, cell-cell infection, Wnt signaling, MAPK signaling, Interferon Signaling.

4. Abstract- It should reflect the summary of the study with 1-2 introductory lines, objective statement, brief methodology, key results, and concluding remarks. This draft seems ambiguous that lacks clarity.
5. Abstract- Avoid abbreviations right away (E.g., WT1). Mention abbreviation in a full form at the first place it appears.

Thank you for your suggestions on the abstract, we have clarified it accordingly. WT1 transcription factor is the official full gene name of the WT1 gene, so we were unable to avoid it, but we have replaced MAPK with ‘mitogen-activated protein kinase’ to avoid abbreviation as suggested.

6. Introduction- This section reads incompetent to establish the study. It should contain disease burden, problem statement, and present approaches to address it. It also should introduce the biochemical factors and their significance in disease progression. For example- What is the rational of acetylation in COVID-19 pathogenesis? This section can be concluded with a paragraph emphasizing approach or plan of present study.

We thank the reviewer for this suggestion, and we revised the introduction accordingly.

7. Results- Figure 1(A) claims to depict the change in H3K27 acetylation in infected cells as compared to uninfected cells. In reality, the figure does not show the data points of acetylation in uninfected cells.

The figure depicts the fold change between infected and uninfected. We apologize for the unclarity, and have now revised the legend to clarify this point.

8. Results- Figure 2 conveys the association between acetylation gain and gene upregulation. However, sub-figures (B, C, G, and H) can be represented with bar graph after percent normalization for ease of understanding.

We replaced the Venn diagrams with bar plots as suggested.

9. Results- A table of pathway regulators is recommended instead of mentioning them all in the text.

We added table 2 which summarizes these results as suggested.

10. Results- For figure 4 (C and F), refer comment No. 8.

We replaced the Venn diagrams with bar plots as suggested.

11. Discussion- This section reads conclusion instead of discussion. This section should discuss the obtained results at the background of available literature. It should identify present research gaps and comment how your observations helped to fill those gaps.

We have now expanded both the discussion on our results and the conclusion, see response to the next comment.

12. Conclusion- This section is major missing.

According to the guide to authors there is no separate section for conclusions and therefore we have merged the discussion and conclusion section to a single section. As the reviewer suggested we have now expanded both the discussion on our results and the conclusion.

13. Methodology- Explain the rationale behind every method that was followed.

We added explanations on the rational of the methods used (see revised methods section).

14. Methodology- It contains the word ‘we’ consistently. Rephrase the sentences and avoid first-person narrative.

We have revised the Methods section to avoid first-person narrative.

15. General- Authors are supposed to be well-versed with the research in this area. The study should establish a rationale or hypothesis properly. There is a great scope of improvement in scientific writing, development of logical methodology, data representation and interpretation.

Reviewer #2: Dear Authors,
The research article under consideration is a nice research work which would prove very useful with challenges we are facing with the pandemic and post pandemic issues. However, this manuscript needs some corrections and revisions as listed below.

We appreciate the interest from the reviewer in our study on the cis regulatory regulation involved in SARS-CoV-2 infection. We have earnestly tried to address the concerns raised and the revised manuscript is more developed and clearer thanks to the reviewer comments.

The title could be simplified for better understanding, may be avoid more technical terms.

Thank you for your suggestion, we simplified the title to “SARS-CoV-2 infection perturbs enhancer mediated transcriptional regulation of key pathways”

The abstract need revision, it need to reflect the contents and the findings.

Thank you for pointing this out, we revised the abstract accordingly.

Introduction and methodology needs to be more comprehensive.

We have extended the introduction and method sections as suggested.

Please correct grammatical and topological errors.

We have corrected the grammar throughout the text.

A takeaway message in form of conclusion would be very helpful.

Thank you for this suggestion, we have added an additional concluding remark to the last paragraph of the 'discussion and conclusion' section.

Thank you

Reviewer #3: Dear authors, I found your draft quite interesting to read and I really loved doing this. However, there are some concerns I couldn’t accept in the present form. I am summarizing few of them below; I hope this would help you for further refinement of your draft.

We thank the reviewer for his thorough review and his interest in the draft.

• Statement “Despite extensive studies of the effects of SARS-CoV-2 infection, we still lack an understanding of the downstream epigenetic and transcriptional alterations” ; however, few information is available- (doi: 10.1016/j.virusres.2022.198853; <https://doi.org/10.1186/s13073-022-01137-4>; <https://doi.org/10.1186/s13148-020-00946-x>) I think you need to compare your findings with these evidences

We thank the reviewer for pointing these papers out. We have clarified our claim, and now state that we lacked understanding of downstream epigenetic and regulatory alterations in the infected cells. The paper by Godoy-Tena et al. indeed studies epigenetic and transcriptomic alterations, but in the monocytes as part of the immune response, and not in the infected lung cells. The paper by Atlante et al. reviews the relevant background and the impact of other SARS viruses, but does not study the impact of SARS-CoV-2 on enhancers in infected lung cells. We have now referred to these works in the text (lines 56-63).

• Figure 1 (b and c) can be revised. I feel you have provided the default images; however, it looks inadequate and can be presented in simpler way

Figure 1 b&c show the results of the motif analysis, and we are therefore also displaying the motifs that were found in the analysis. To be clearer and more comprehensive the motifs are also simply listed in Supplementary Table S2.

• In table you mention “lower and higher acetylation levels”; what defines this? Is there any threshold to mention whether the acetylation is higher or lower?

We apologize for the unclarity. The table refers to the enhancers previously described as gaining or losing acetylation (e.g. requiring FDR<0.05, fold-change > 150%). We have now clarified the table legend.

• Please avoid providing any references in the results section

We believe that it is important to give credit and provide references when it is due.

• All the Venn diagrams should be revised; the area containing higher items should be larger than another; please revise

Unfortunately, due to the large differences in group sizes, a proportional Venn diagram would be difficult to read, we have therefore replaced all Venn diagrams with bar plots.

• I see your study focuses towards Wnt signaling, MAPK signaling and pro-inflammatory which are also regulated by IFN1. Just out of curiosity, did you found any epigenetic and transcriptional alterations in IFN1 signaling (Jak-STAT)? During viral infection, there is modulation of these pathways to produce vicious cycle in response to infection.

Indeed, differentially expressed genes near differential enhancers are strongly enriched in interferon signaling (Supplementary Table S4), as well as when focusing on genes near WT1 enhancers (Supplementary Table S6). In addition, the STAT motif is enriched in enhancers that gained H3K27ac. We did not specifically mention them as they are contained in more general pathways such as “innate immune response” and “regulation of cytokine-mediated signaling pathway”. However, this comment made us realize we should have included them explicitly and we have now added them (revised figures 3 and 5).

• Figure 3; “Others….” Please name some in foot note of the figure legend

We apologize for the confusing terms. The ’other’ category simply represent that the listed pathways on the left do not fall to a specific category, but all the pathways are listed on the left. We have now removed the ‘other’ category for clarity.

• Please rewrite all the statements in past tense in results and methodology

We have rephrased the text as suggested.

• Few errors can be observed in phrase and sentences; Please revise

We went through the entire manuscript and revised accordingly.

• Figure 4(g) should be revise; GO terms are too close to read

Thank you for your comment. To make the figure more legible we have split it to two figures, see revised Figure 5.

• In methodology; please detail “Acetylation distribution”

We apologize for the unclarity. We rewrote this subsection (lines 416-420).

• In methodology, “we defined an enrichment fold change score by (target gene in term * total genes)/(total target genes * gene in term)”; How do you define this? Can you provide a reference for it?

We apologize for the confusing terms. We simply counted the number of target genes in term, the numbers of genes in term, the total target genes and the total number of genes, and then calculated (target gene in term * total genes)/(total target genes * gene in term). We refer to this number as ‘enrichment fold change score’. We have now clarified the text (lines 426-427).
