# Supplementary material for: SARS-CoV-2 infection perturbs enhancer mediated transcriptional regulation of key pathways
Source: PLoS Comput Biol. 2023 Aug 10;19(8):e1011397. doi: 10.1371/journal.pcbi.1011397 (PMC10443870; doi:10.1371/journal.pcbi.1011397)
Supplement: Attachment — Submitted filename: rebuttal2.docx [file pcbi.1011397.s009.docx]

**RESPONSE TO REVIEWER COMMENTS**

We are very grateful for all the reviewers’ time and thoughtful comments and feedback.

Reviewer #3: I am still puzzled with enrichment fold change score calculation? You responded in line 426-427 but what is the basis for that? Please quote this calculation if not please detail the rationale for this formula for enrichment fold change score.

We apologize for the continuous unclarity. We are now citing Zeeberg et al. (ref 31) for a popular gene set enrichment method that uses relative enrichment, and have changed the name of the score to ‘relative enrichment’ to match Zeeberg et al.

I do not agree on your statement “We believe that it is important to give credit and provide references when it is due” for my query “Please avoid providing any references in the results section”. The result section in the manuscript (research) is only meant to provide the findings of your methodology hence it is called results section. I did not mean not to give the credit but it should be in the order. It would be better you could discuss the finding and quote the statement only in discussion section not in results.

Thank you for clarifying. We have moved all the references from the Results section to the Methods and Discussion sections.
